# Biallelic pathogenic variants of PARS2 cause developmental and epileptic encephalopathy with spike‐and‐wave activation in sleep

**DOI:** 10.1002/mgg3.2311

**Published:** 2023-12-13

**Authors:** Laura Licchetta, Lucia Di Giorgi, Margherita Santucci, Lisa Taruffi, Carlotta Stipa, Raffaella Minardi, Valerio Carelli, Francesca Bisulli

**Affiliations:** ^1^ IRCCS Istituto delle Scienze Neurologiche di Bologna Full member of the European Reference Network EpiCARE Bologna Bologna Italy; ^2^ Department of Biomedicine, Neuroscience and Advanced Diagnostics University of Palermo Palermo Italy; ^3^ Department of Biomedical and Neuromotor Sciences University of Bologna Bologna Italy

**Keywords:** continuous spikes and waves during slow sleep (CSWS), developmental and epileptic encephalopathy (DEE), *PARS2*, spike‐and‐wave activation in sleep (SWAS)

## Abstract

**Background:**

Biallelic pathogenic variants in the mitochondrial prolyl‐tRNA synthetase 2 gene (*PARS2*, OMIM * 612036) have been associated with Developmental and Epileptic Encephalopathy‐75 (DEE‐75, MIM #618437). This condition is typically characterized by early‐onset refractory infantile spasms with hypsarrhythmia, intellectual disability, microcephaly, cerebral atrophy with hypomyelination, lactic acidemia, and cardiomyopathy. Most affected individuals do not survive beyond the age of 10 years.

**Methods:**

We describe a patient with early‐onset DEE, consistently showing an EEG pattern of Spike‐and‐Wave Activation in Sleep (SWAS) since childhood. The patient underwent extensive clinical, metabolic and genetic investigations, including whole exome sequencing (WES).

**Results:**

WES analysis identified compound heterozygous variants in *PARS2* that have been already reported as pathogenic. A literature review of *PARS2*‐associated DEE, focusing mainly on the electroclinical phenotype, did not reveal the association of SWAS with pathogenic variants in *PARS2*.

Notably, unlike previously reported cases with the same genotype, this patient had longer survival without cardiac involvement or lactic acidosis, suggesting potential genetic modifiers contributing to disease variability.

**Conclusion:**

These findings widen the genetic heterogeneity of DEE‐SWAS, including *PARS2* as a causative gene in this syndromic entity, and highlight the importance of prolonged sleep EEG recording for the recognition of SWAS as a possible electroclinical evolution of *PARS2*‐related DEE.

## Graphical abstract

We first describe a patient with early onset developmental and epileptic encephalopathy and a pattern of spike‐and‐wave activation in sleep (DEE‐SWAS) since childhood. WES analysis identified biallelic pathogenic variants in PARS2. Our findings expand the genetic heterogeneity of DEE‐SWAS to include PARS2 as a causative gene and highlight the importance of prolonged sleep‐EEG recording for recognition of SWAS as an electroclinical evolution of PARS2‐DEE.

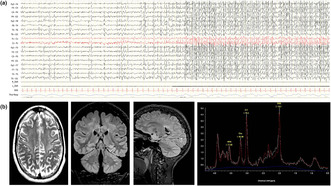

## 1 INTRODUCTION

The *PARS2* gene (OMIM * 612036) is a nuclear gene encoding for the mitochondrial prolyl‐tRNA synthetase 2, an aminoacyl‐tRNA synthetase essential for mitochondrial protein biosynthesis and oxidative phosphorylation (Almuqbil et al., 2020). Biallelic pathogenic variants of *PARS2* are associated with Developmental and Epileptic Encephalopathy‐75 (DEE‐75, MIM #618437), a multisystem mitochondrial disorder, characterized by microcephaly, dysmorphic features, hypotonia, intellectual disability (ID), epileptic spasms, cerebral atrophy with delayed myelination, lactic acidemia and cardiomyopathy. Less common epileptic manifestations are focal seizures, myoclonus and generalized seizures. Most patients die within the first 10 years of life (Almuqbil et al., 2020). In these patients, the EEG usually shows multifocal spikes and hypsarrhythmia, consistent with a clinical diagnosis of Infantile Epileptic Spasms Syndrome (IESS).

Spike‐and‐wave activation in sleep (SWAS) is an EEG pattern typical of a spectrum of epileptic encephalopathies (EE) or DEE characterized by regression in cognitive, behavioral, or psychiatric function and motor deterioration with dyspraxia or dystonic features, recently renamed EE‐SWAS or DEE‐SWAS (Specchio et al., 2022). SWAS is characterized by a significant increase in slow spike‐and‐wave complexes at 1.5–2 Hz, particularly during stage N2 of sleep. This abnormal activity is typically diffuse, but may be more localized, often in the frontal regions, or multifocal. Patients usually present with infrequent, focal, drug‐responsive seizures between the ages of 2 and 5 years. As the disease progresses, multiple seizure types tend to appear. These include focal seizures with or without impaired awareness, typical and atypical absences, atonic seizures, and focal motor seizures with negative myoclonus. Some patients may present only acquired aphasia without experiencing clinical seizures (Specchio et al., 2022).

DEE‐SWAS may be caused by a variety of etiologies, including brain structural abnormalities and genetic factors inherited in either monogenic or complex patterns. Up to 50% of patients have a family history of seizures (Tsai et al., 2013). *GRIN2A* (OMIM *138253), encoding the alpha 2 subunit of the N‐methyl‐D‐aspartate (NMDA) glutamate receptor, is the major monogenic factor. Pathogenic variants in this gene have been associated with a range of DEE‐SWAS with varying degrees of severity, included in the epilepsy‐aphasia spectrum disorders (Carvill et al., 2013; Lesca et al., 2019).

We describe the case of a 25‐year‐old woman with DEE and an EEG pattern of SWAS carrying biallelic variants in *PARS2*. We also performed a literature review of all the reported cases of *PARS2*‐associated DEE.

## 2 MATERIALS AND METHODS

The patient was referred to the Epilepsy Center of our Institute after the transition from pediatric to adult care. She underwent an accurate clinical, neurophysiological and neuroradiological assessment. Whole exome sequencing (mean coverage: 52X; read length: 150 bp) was performed in the context of the Epi25 Project (Epi25 Collaborative, http://epi‐25.org) for which she was recruited in 2017, after obtaining specific consent. The study was approved by the local ethics committee (CE 16057). Details of variant analysis, interpretation and validation were provided elsewhere (Minardi et al., 2020).

A systematic search to map the literature with regards to *PARS2*‐related DEE was performed between March and April 2023. The PubMed database was searched using a combination of the key words “PARS2” AND “seizures” OR “epilepsy” OR “encephalopathy”, with no restrictions on publication date or language. The articles cited in the retrieved papers were also screened for additional references. The full text of the original articles was carefully reviewed for relevant content and for an in‐depth characterization of the epilepsy phenotype, focusing mainly on the possible association of SWAS.

## 3 RESULTS

### 3.1 Case presentation

This 25‐year‐old woman, born from unrelated parents, presented with epileptic spasms and hypsarrhythmia at 5½ months of age. Since then, regression and severe psychomotor developmental delay were evident. She was diagnosed with IESS and started on vigabatrin and valproate with seizure control and EEG normalization. The electroclinical amelioration coincided with a slow progression of motor milestones. However, there was no improvement in cognitive functioning, and structured verbal language was never acquired, except for the repetition of a few words at the age of 3. Neurologic examination (NE) showed microcephaly, profound ID, hand stereotypies, and spastic paraparesis, treated with botulinum toxin at age 2–4 years. Subsequent EEG showed multifocal epileptiform abnormalities, enhanced by sleep. Brain MRI at the age of 3 years showed mild cortical atrophy, white matter signal alteration in the bilateral subcortical retrotrigonal region suggestive of incomplete maturation, and a thinning of the corpus callosum.

From the age of 8 years, there was a progressive deterioration of the EEG, which evolved into a SWAS pattern. Neuropsychological assessment at that stage documented regression, with a complete loss of language ability. Brain MRI findings were stable. Despite the absence of obvious clinical events, levetiracetam and clonazepam were added to valproate, without improvement in the EEG.

At the age of 16, she had a seizure relapse with two convulsive seizures two hours apart, each lasting less than one minute. On that occasion, a decrease in valproate plasma levels was found. The EEG recordings consistently showed a pattern of SWAS. Brain MRI showed a greater extent of the bilateral and symmetric white matter hyperintensity in Flair, involving in addition to the retrotrigonal regions and semi‐oval centers, the subcortical white matter of the pre‐ and post‐central gyri.

**FIGURE 1:**
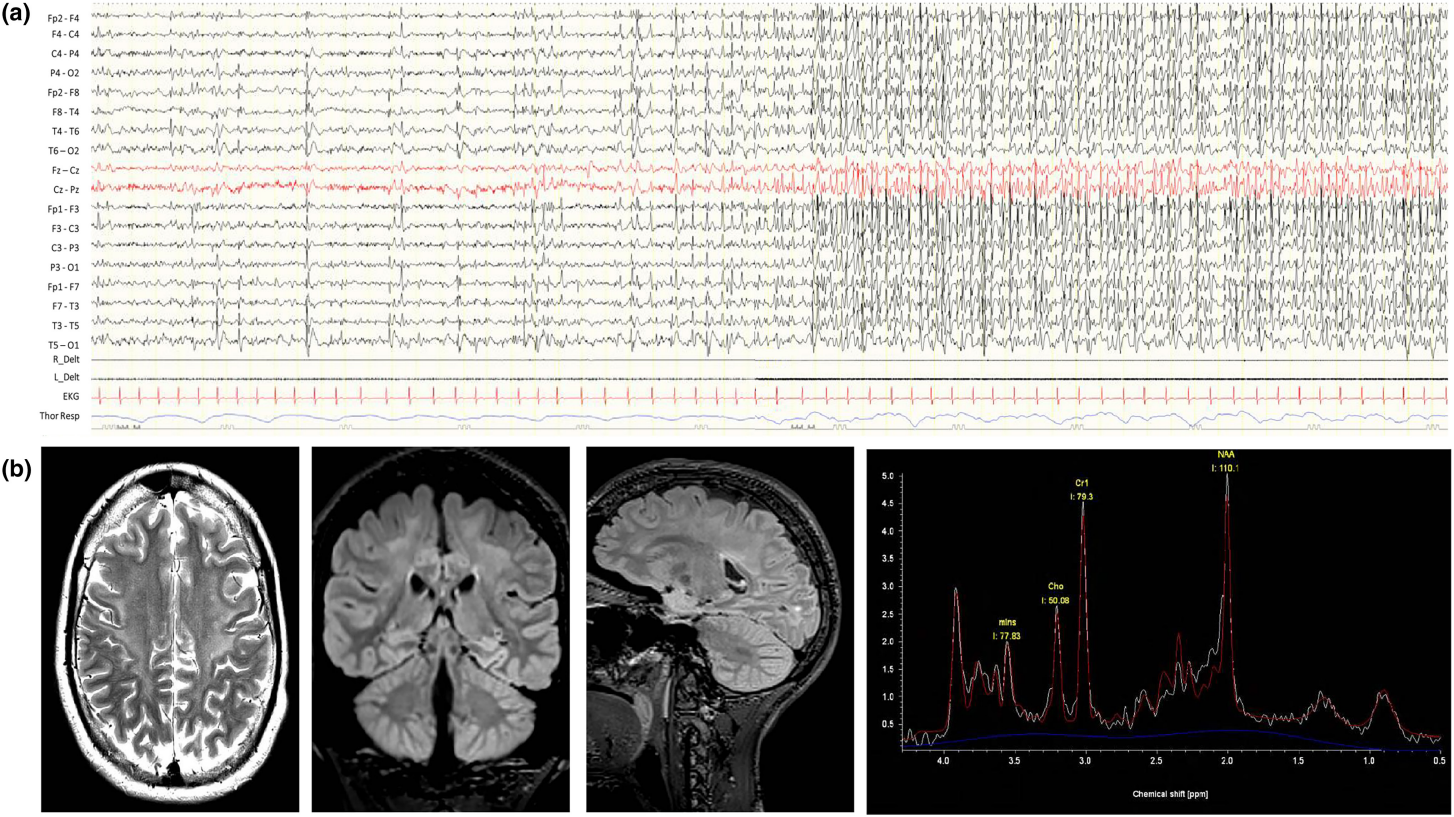
(a) EEG (21 years old): during drowsiness, bilateral, synchronous, and asynchronous spike–wave (SW) over the temporal–parietal regions enhanced by sleep, evolving to a pattern of continuous SW during sleep (SWAS). (b) Brain MRI (21 years old): diffuse, bilateral hyperintensity in T2‐FLAIR of the subcortical white matter (retrotrigonal regions, semioval centers, pre and post‐central gyrus) with reduced apparent diffusion coefficient (ADC), suggestive of diffuse hypomyelination. Posterior atrophy. The spectroscopy study was performed by placing two volumes on the right, in the altered white matter of the Rolandic region and in the morphologically apparently free over the frontal region. The results show a modest reduction in NAA/Cr and Cho/Cr ratios.

Since the age of 18, she has experienced brief episodes of abrupt bilateral stretching of the oral rhyme (a kind of facial grimace) and head flexion, vocalization, facial blushing, and sometimes urinary incontinence compatible with subtle spasms. These events, occurred in clusters mainly on awakening, and lasted from 5 to 20 min, multiple times per day. A transient improvement was achieved with the optimization of valproate therapy. Over the years, she was treated with several other anti‐seizure medications (ASMs) such as ethosuximide, acetazolamide, zonisamide and rufinamide. Only after steroid therapy at the age of 21 (cycles of intravenous methylprednisolone for three consecutive days/month for six months) did the seizures and EEG pattern of SWAS cease for several months. Currently, she has been experiencing multiple seizures per day despite a combination of three ASMs (valproate, lamotrigine and clobazam). The EEG still shows frequent epileptiform abnormalities during drowsiness that became continuous in sleep (Figure 1a).

Brain MRI and proton magnetic resonance spectroscopy at the age of 21 years showed diffuse hypomyelination and atrophy, stable compared with the previous one, and a modest reduction in the NAA/Cr and Cho/Cr ratios (Figure 1b).

The patient underwent extensive metabolic and cardiological exams, which were unremarkable. Genetic investigations including karyotype, PCR and fluorescence in situ hybridization for X‐fragile, CGH‐array, direct sequencing of *MECP2*, *CDKL5*, *FOXG1*, *PCDH19, SLC2A1*, and a multigene epilepsy panel, were all negative. At the age of 21 years, she underwent WES analysis that showed compound heterozygous variants in *PARS2* (NM_152268.3): c.1091C>G (p.Pro364Arg) and c.283G>A (p.Val95Ile), inherited from the healthy father and mother, respectively, and already reported in the literature to be pathogenic (Table 1).

**TABLE 1:** Clinical and genetic findings in *PARS2*‐related DEE: our case and reports from literature review.

	Thisstudy	Sofou et al. (2015)	Pronicka et al. (2016)	Mizuguchi et al. (2017)		Ciara et al. (2018)			Yin et al. (2018)		Al Balushi et al. (2020)	Maddirevula et al. (2019)	Almuqbil et al. (2020)
Gender, age	F, 25y	M, †2.2y	M, †8.5y	F, 9 y	F, 3 y	M, †8.5y	F, †8 y	M, 6.5 y	F, 3 y	F, †4 m	F, 3.3 y	na	M, †21 y
ID	+	+	+	+	+	+	+	+	+	+	+	+	+
Seizure onset	5.5 m	2.5 m	Neonatal	5 m	4 m	4 m	5 m	4 m	9 m	3 m	4 m	na	8 m
Seizure type	IS	IS, F to bilT‐C	na	IS, F	IS	IS, My	IS, T‐C, My	IS, My	IS	IS	IS	na	IS, G
Neurological examination	MC, Hyp,Pyr, Aph	MC, Hyp, Dyst, Vis	MC	MC, Hyp Aph	MC, Pyr	MC, Hyp, Pyr, Aph Vis	MC, Hyp Pyr, Aph, Vis	MC, Hyp Pyr, Aph, Vis	MC, Hyp, Dyst	MC, Hyp, Dysph	MC, Cereb, Pyr, Dyst	na	MC, Hyp, Aph
EEGepileptiform anomalies	Hyps, Mf, SWAS	Hyps, bil>post	na	Hyps	Mf>post	Hyps; G; Mf	Hyps; G; Mf	Hyps; G; Mf	Hyps; Mf	Hyps; Mf; asym BA	na	na	Hyps
Brain MRI	A, Hypom	A (CT), BG^a^	BG	A>f, Hypom	A>f, Hypom	A f, CC, Hypom, stroke‐like les, BG	A f, CC, Hypom	A f, CC, Hypom	A>f, Hypom, Cereb les	Cereb les	A, CC	A	A f‐p, BG, thalami les
Cardio‐myopathy	−	+	+	na	na	+	+	+	−	na	−	na	+
Lactate level (blood/CSF)	N/na	↑/↑	na	↑/↑	↑/na	↑/N	N/N	na/N	↑/na	↑/na	N/na	N/na	N/na
Muscle biopsy (biochemical/morphology)	na	↓ activity complex I, IV/N	N/N	na	na	↑ citric synthase activity/N	na	na	na	na	na	na	na
Genotype (aa change)	**V95I; P364R**	K378fs1; S279L	**P364R**; I80T	**V95I**; E203K	**V95I**; E203K	**P364R**; I80T	**P364R**; I80T	**P364R**; I80T	**V95I**; R202G	**V95I**; R202G	**V95I; P364R**	**V95I**; **V95I**	**V95I; P364R**
Treatment/drug‐resistance	VPA, LTG, CLB, other ASMs, steroids/+	PB, CNZ/na	ASMs/na	ASMs/+	ACHT/−	GVG, VPA, ACHT, LTG, TPM, CLB /+	na/−	LEV, CLB, GVG/na	TPM, VPA/−	ACTH/na	na/na	na/na	ACTH, ASMs/−

*Note*: In Bold: *PARS2* variants found in our patient either in the same combination or associated with a different one.

Abbreviations: A, atrophy; Aph, aphasia; ASMs, antiseizure medications, unspecified; Asym, asymmetric; BA, Background activity; BG, basal ganglia lesions; Bil, bilateral; CC, corpus callosum thinning; Cereb, cerebellar; CLB, clobazam; CNZ, clonazepam; CSF, cerebrospinal fluid; CT, computed tomography scan; Dysp, Dysphagia; Dyst, dystonia; f, frontal; F, female; F to bilT‐C, focal to bilateral tonic–clonic seizures; G, generalized; GVG: vigabatrin; Hyp, hypotonia; Hypom, Hypomyelination; Hyps, Hypsarrhythmia; ID, intellectual disability; IS, infantile spasms; les, lesions; LEV, levetiracetam; LTG, lamotrigine; M, male; m, months; MC, microcephaly; Mf, multifocal abnormalities; My, myoclonic; N, normal; na, not available/not known; p, parietal; PB, phenobarbital; post, posteriorly; Pyr, Pyramidal signs (including limbs hypertonia); T‐C, tonic–clonic seizure; TPM, topiramate; Vis, visual impairment; VPA, valproate; y, years; †, deceased; >, prevalent.

^a^ BG necrotic lesion documented by autopsy.

### 3.2 Literature review

The search of the PubMed database yielded eight records corresponding to 12 patients with DEE‐75 (Al Balushi et al., 2020; Almuqbil et al., 2020; Ciara et al., 2018; Maddirevula et al., 2019; Mizuguchi et al., 2017; Pronicka et al., 2016; Sofou et al., 2015; Yin et al., 2018). All electro‐clinical, neuroradiological and genetic data are summarized in Table 1. None of the reported cases with *PARS2*‐DEE had an EEG pattern of SWAS.

## 4 DISCUSSION

We reported on a patient with DEE‐SWAS carrying compound heterozygous pathogenic variants in *PARS2*. The literature review did not reveal other cases of *PARS2*‐related DEE‐SWAS.

In addition to *GRIN2A*, genes associated with DEE‐SWAS to date include, in order of frequency, *CNKSR2, ZEB2, KCNA2, KCNQ2, SLC9A6, SCN2A, KCNB1*, *SLC6A1, WAC, TET3, KCND2, KCNA1, ATP1A3, MECP2, SERPINI, TUBA1A, CDKL5, KANSL1, ATN1*, *SRPX2*, *OPA3*, *HIVEP2* (Freibauer et al., 2023; Kessi et al., 2018), *IL1RAPL1* (Jiang et al., 2022), *FRRS1L* (Mir et al., 2023), *CARS2* and *RARS2* (Freibauer et al., 2023). The latter two are other nuclear genes encoding different mitochondrial aminoacyl‐tRNA synthetases, cysteinyl and argynil, respectively.

Our patient initially presented with IESS with hypsarrhythmia, one of the typical presentations of DEE‐75. After a period of electroclinical stability, her EEG pattern changed in late childhood, showing a progressive increase in epileptiform abnormalities during sleep to continuous SWAS. It is known that DEE with hypsarrhythmia can progress to DEE‐SWAS (Mir et al., 2023), an age‐dependent syndrome that typically occurs between the age of 4 and 8 years, but this has never been reported in *PARS2*‐related DEE. This may be related to the short survival of the patients carrying pathogenic variants of *PARS2* reported to date, most of whom died in childhood, before possibly developing a SWAS pattern. Nevertheless, no SWAS pattern was described in the older patient reported (Almuqbil et al., 2020), even though no sleep EEG recording was mentioned among the investigations performed.

The pathogenic variants identified in our patient were already reported in the literature, either together in compound heterozygosity (Al Balushi et al., 2020; Almuqbil et al., 2020) or individually in combination with other pathogenic variants (Ciara et al., 2018; Mizuguchi et al., 2017; Pronicka et al., 2016; Yin et al., 2018) or in homozygosity (Maddirevula et al., 2019) (Table 1).

Compared with the cases with identical genotypes, our patient showed a similar survival to the case reported by Almuqbil and co‐authors who is the only one who died in adulthood (Almuqbil et al., 2020), at the age of 21 years due to cardiac dysfunction. Cardiac involvement is commonly observed in patients with pathological variants of *PARS2*, usually with initial hypertrophic cardiomyopathy followed by a dilatative evolution in a brief time before death (Almuqbil et al., 2020) usually occurring in childhood (Ciara et al., 2018; Sofou et al., 2015). No similar findings were observed in our patient, in whom the cardiac assessment repeated at 25 years of age was normal. Thus, the absence of cardiac involvement may be the reason for her long survival compared to other *PARS2*‐mutated patients. In addition, unlike the aforementioned case (Almuqbil et al., 2020), our patient did not have recurrent infections or liver involvement.

A severe global developmental delay with ID and “absent speech” characterized most of these cases. Interestingly, our patient experienced a further cognitive regression with complete loss of language after developing SWAS. Notably, this EEG pattern may characterize conditions with subacute onset of acquired aphasia without or only with infrequent and self‐limiting seizures (Specchio et al., 2022). In addition to microcephaly and axial hypotonia, which are the most common findings in NE in *PARS2*‐DEE, our patient showed spastic paraparesis with significant gait impairment. Pyramidal signs (including limb hypertonia and Babinski's sign, possibly in combination with axial hypotonia) have been observed also in patients with the same pathogenic variants (Yin et al., 2018), as well as in patients with different genotypes (Ciara et al., 2018; Mizuguchi et al., 2017).

Brain MRI in our patient shows progressive diffuse atrophy and hypomyelination, one of the most common neuroradiological findings in *PARS2‐*related DEE (Table 1). She did not show basal ganglia (Almuqbil et al., 2020; Ciara et al., 2018; Pronicka et al., 2016; Sofou et al., 2015) thalami (Almuqbil et al., 2020), or cerebellum (Yin et al., 2018) involvement, which have been occasionally reported in other cases, nor stroke‐like lesions described only anecdotally (Ciara et al., 2018).

Although high lactate levels and myopathic changes on muscle biopsy are frequently reported in this mitochondrial DEE, our case, as well as patients with the same genotype, did not show laboratory alterations or lactate accumulation on MRI spectroscopy (Table 1). As for other adult cases of DEE, the application of next‐generation sequencing techniques in our patient allowed genetic diagnosis decades after the onset of epilepsy, ending a long diagnostic odyssey. Genetic diagnosis also led to improved patient management through surveillance and monitoring of possible comorbidities. In terms of therapy, whereas valproate is contraindicated in other mitochondrial encephalopathies, such as Alpers syndrome due to pathogenic variants in *POLG* (DNA polymerase γ), as it can lead to fulminant liver failure (Pronicka et al., 2011), there are no definite data on valproate toxicity in *PARS2*‐related DEE. In Ciara and coauthors, a patient with different compound heterozygosity of *PARS2* was treated with valproic acid, which appeared to be associated with clinical worsening and liver failure (Ciara et al., 2018). In our patient, however, valproate administration coincided with a period of seizure freedom and later with a transient reduction in seizure frequency, suggesting that valproate treatment is not an absolute contraindication in mitochondrial disorders other than *POLG*‐related ones. Steroid pulse therapy may be a treatment option for *PARS2*‐related DEE‐SWAS. In fact, several studies on the effect of steroids and ACTH at different dosage protocols in SWAS‐related conditions have shown a transient positive response to steroids with normalization of the EEG and improvement of neuropsychological functions, in particular language and behavior (Veggiotti et al., 2012). Despite the temporary resolution of SWAS with corticosteroids, our patient eventually had a seizure relapse with persistence of SWAS into adulthood. The typical SWAS is a self‐limited condition that usually resolves by adolescence. The longer duration of this pattern in our patient may be related to the underlying genetic etiology, responsible for mitochondrial encephalopathy with a progressive pattern of hypomyelination on brain MRI. In fact, several studies have shown that the duration of SWAS or EEG abnormalities is significantly longer in symptomatic/structural cases (Caraballo et al., 2013) or in patients with an unfavorable outcome in whom symptomatic/structural etiologies were more common (Öztoprak et al., 2021). Furthermore, in the childhood cases reported in the literature, the persistence of the SWAS pattern may have been missed due to the lack of electroclinical follow‐up.

In conclusion, we first reported *PARS2* as a new gene determining DEE‐SWAS. The recognition of SWAS as a possible electroclinical evolution of *PARS2*‐related DEE will help in the diagnosis and appropriate management of these patients. Moreover, it underlines the importance of a complete neurophysiological assessment including prolonged and serial sleep EEG recordings. Prolonged survival, absence of lactic acidosis and cardiac involvement, and transient electro‐clinical improvement with valproate are additional atypical features in our case. Our findings widen the spectrum of genes associated with DEE‐SWAS, to include *PARS2*.

## AUTHOR CONTRIBUTIONS

*Patient phenotyping/clinical analysis*: Laura Licchetta, Margherita Santucci, Carlotta Stipa. *Molecular genetic/bioinformatic analysis*: Laura Licchetta, Raffaella Minardi. *Literature revision*: Lucia Di Giorgi, Lisa Taruffi, Laura Licchetta. *Wrote manuscript*: Laura Licchetta, Lucia Di Giorgi, Margherita Santucci. *Reviewed manuscript*: Francesca Bisulli, Valerio Carelli.

## FUNDING INFORMATION

Italian Epilepsy Federation (FIE), project "Identification of genes underlying early‐onset epileptic encephalopathies" to F. Bisulli

## CONFLICT OF INTEREST STATEMENT

None of the authors have any conflict of interest to disclose.

## ETHICS STATEMENT

We confirm that we have read the Journal's position on issues involved in ethical publication and affirm that this report is consistent with those guidelines.

## Data Availability

Data are available on request.
